# Progressive Depletion of Rough Endoplasmic Reticulum in Epithelial Cells of the Small Intestine in Monosodium Glutamate Mice Model of Obesity

**DOI:** 10.1155/2016/5251738

**Published:** 2016-06-29

**Authors:** Kazuhiko Nakadate, Kento Motojima, Tomoya Hirakawa, Sawako Tanaka-Nakadate

**Affiliations:** ^1^Department of Basic Science, Educational and Research Center for Pharmacy, Meiji Pharmaceutical University, Tokyo 204-8588, Japan; ^2^Faculty of Pharmaceutical Sciences, Meiji Pharmaceutical University, Tokyo 204-8588, Japan; ^3^Department of Pharmacology and Toxicology, Dokkyo Medical University School of Medicine, Tochigi 321-0293, Japan

## Abstract

Chronic obesity is a known risk factor for metabolic syndrome. However, little is known about pathological changes in the small intestine associated with chronic obesity. This study investigated cellular and subcellular level changes in the small intestine of obese mice. In this study, a mouse model of obesity was established by early postnatal administration of monosodium glutamate. Changes in body weight were monitored, and pathological changes in the small intestine were evaluated using hematoxylin-eosin and Nissl staining and light and electron microscopy. Consequently, obese mice were significantly heavier compared with controls from 9 weeks of age. Villi in the small intestine of obese mice were elongated and thinned. There was reduced hematoxylin staining in the epithelium of the small intestine of obese mice. Electron microscopy revealed a significant decrease in and shortening of rough endoplasmic reticulum in epithelial cells of the small intestine of obese mice compared with normal mice. The decrease in rough endoplasmic reticulum in the small intestine epithelial cells of obese mice indicates that obesity starting in childhood influences various functions of the small intestine, such as protein synthesis, and could impair both the defense mechanism against invasion of pathogenic microbes and nutritional absorption.

## 1. Introduction

Obesity has recently become a major cause of the metabolic syndrome worldwide [1, 2]. Moreover, the incidence of obesity has more than doubled in children and quadrupled in adolescents in the past 30 years [3]. Obese children and adolescents are likely to be obese as adults; therefore, they are at an increased risk of adult health problems, such as heart disease, cardiovascular disease, type 2 diabetes, stroke, and osteoarthritis [4–8]. Chronic obesity is also associated with an increased risk of many types of cancer, including cancer of the breast, colon, endometrium, esophagus, kidney, pancreas, gall bladder, thyroid, ovary, cervix, and prostate as well as multiple myeloma and Hodgkin's lymphoma [9]. Furthermore, metabolic syndrome is a major predictor of nonalcoholic fatty liver disease (NAFLD) [10, 11]. In NAFLD, a steadily progressive disease, the findings of hepatitis and fibrosis and changes in hepatocytes are similar to those seen in alcoholic liver disease [12]. Many patients with NAFLD are obese and have comorbid hyperlipidemia, hypertension, and insulin resistance. Therefore, NAFLD is now recognized as the hepatic manifestation of metabolic syndrome [13–15]. The pathogenesis of NAFLD remains unclear. However, it is thought that inflammation and fibrosis in the liver are caused by a two-phase process [16]. The first step involves an increase in fatty acid biosynthesis or blockade of fatty acid combustion, and the second step involves an increase in factors, such as oxidative stress and lipid peroxidation. In terms of an immunological pathogenesis, it has been suggested that lifestyle factors, such as overeating or unbalanced diet, alter the quality and/or quantity of bacterial flora in the intestine. This can result in the translocation of Gram-negative bacteria from the gut into the portal vein, which activates resident liver macrophages (Kupffer cells), setting up an immune response.

The intestinal mucous membrane epithelium has a defense mechanism to prevent the invasion of pathogenic microbes such as bacteria. When the permeability of this intestinal epithelium is increased by excessive alcohol intake or overeating, the defense mechanism can fail [17]. Liver damage is caused by an inflow of bacteria and endotoxins through the lining of the intestinal tract into the bloodstream, including the portal vein. Therefore, failure of the defense mechanism of the intestinal mucous membrane is very important in the pathogenesis of various chronic liver diseases [17, 18]. The transport of material through the intestinal epithelium is either transcellular via the intracellular pathway, or paracellular via the intercellular pathway. The paracellular pathway is involved in the absorption of minerals such as calcium and is controlled by an intercellular barrier. The transcellular pathway is involved in the intake of nutrients and is controlled by regulation of the many transporters and channels on the membrane of intestinal epithelium cells. Failure of the defense mechanism provided by the intestinal mucous membrane underlies many pathological conditions.

In a previous study of mice with monosodium glutamate- (MSG-) induced obesity, we found that lipid droplets accumulated in the hepatocytes of obese mice and levels of nonesterified fatty acid (NEFA), low-density lipoprotein cholesterol (LDL-C), high-density lipoprotein cholesterol (HDL-C), and triglyceride (TG) were increased compared to controls [19]. Other studies have also reported that lipid droplets accumulate in hepatocytes in chronic obesity and NAFLD [1, 20]. Moreover, our recent scanning electron microscopic analysis of the livers of mice with MSG-induced obesity showed sinusoidal dilatation and swelling of sinusoidal fenestrations [21]. However, little is known about pathological changes in the small intestine associated with chronic obesity. It is possible that obesity leads to functional changes in the small intestine, such as altered nutritional absorption through the epithelial cells into blood vessels. Therefore, we used light and electron microscopy to investigate structural details of the small intestinal epithelium in mice with obesity induced using MSG during the early perinatal period.

## 2. Materials and Methods

### 2.1. Animals

A total of 16 C57BL/6J male mice (Charles River, Japan) were used in this study. The mice were housed under temperature and humidity controlled conditions with a 12 : 12 h light-dark cycle and free access to food and water. All experiments were performed in accordance with the National Institutes of Health Guide for the Care and Use of Laboratory Animals. The animal research committee of Meiji Pharmaceutical University approved the experimental protocol. All efforts were made to minimize the suffering of animals and to reduce the number of animals used in the present study.

### 2.2. Induction of Obesity

As reported in our previous studies, MSG was used to induce obesity [19, 21]. On postnatal days 1, 2, 4, 6, 8, and 10, eight male C57BL/6J mice were intrasubcutaneously injected with MSG (2 mg/g of body weight; Wako Pure Chemical Industries, Ltd., Japan). The other eight male mice were intrasubcutaneously injected with saline on the same days to serve as controls. After weaning, the body weight of each mouse was measured once a week.

### 2.3. Tissue Preparation

At 15 weeks of age, all mice were anesthetized with an overdose of Sodium pentobarbital (50 mg/kg, ip, Nembutal; Abbott Lab., North Chicago, IL), and their digestive system was quickly removed. The total length of small intestine from all animals and the diameter of their small intestines (5 places/animal) were measured, and then the wet volumes of their small intestines were weighted. For histological analysis using light microscopy, a section of the small intestine was immersed in fixative containing 4% paraformaldehyde in 0.1 M phosphate buffer (PB; pH 7.4) for 2 days at 4°C. After fixation, the small intestine was trimmed, washed with PB, dehydrated through graded concentrations of ethanol, cleared in xylene, and embedded in paraffin. For transmission electron microscopy, a section of small intestine was immersed in fixative containing 4% paraformaldehyde and 2% glutaraldehyde in 0.1 M PB for 2 days at 4°C. After washing with PB, the small intestine was immersed in 1% osmium tetroxide solution, dehydrated through graded concentrations of ethanol, saturated in propylene oxide, and embedded in Epon-812 resin (TAAB Co., Switzerland).

### 2.4. Histological Analysis

For hematoxylin-eosin (HE) and toluidine blue (Nissl) staining and to further investigate rough endoplasmic reticulum (rER), Golgi apparatus, and tight junction, the small intestine blocks were cut into 3 *μ*m thick sections on a microtome (REM-710; Yamato, Japan). The sections were mounted on glass slides, deparaffinized with xylene, and immersed in degraded concentrations of ethanol. After washing in distilled water, several sections from each group were stained with HE or Nissl solution. After washing, the sections were dehydrated through graded concentrations of ethanol, cleared with xylene, and cover slipped. The rER and Golgi apparatus in mucosal epithelial cells in the small intestine samples from each group were stained using anti-GRP78 Bip antibody (ab21685, Abcam, Cambridge, MA) or anti-golgin 97 antibody (ab84340, Abcam, Cambridge, MA), respectively. And to identify the tight junction between epithelia, the sections were stained with anti-ZO-1 antibody (gift from Dr. M. Itoh, Department of Biochemistry, School of Medicine, Dokkyo Medical University, Japan). After immunofluorescence procedures, the sections were cover slipped. All images of stained sections were captured using a CCD camera system (BZ-X700; Keyence, Japan).

### 2.5. Electron Microscopic Analysis

All small intestine blocks embedded in Epon-812 resin were trimmed using light microscopy. Several sections (3 *μ*m) were cut with an MT-XL ultramicrotome (Research and Manufacturing Company, Tucson, AZ) and stained with Nissl solution, and images were captured using the CCD camera system (BZ-X700; Keyence). The other sections were checked, reembedded in Epon-812 resin, cut with an MT-XL ultramicrotome into ultrathin sections (70 nm), and picked up on grids (Veco Co., Eerbeek, Holland). The ultrathin sections were stained with electron stain and examined with a conventional transmission electron microscope (JEM-1011; JEOL LTD., Tokyo, Japan), and the images captured using the CCD camera system.

### 2.6. Data Analysis

All images captured using the CCD camera system were measured using the ImageJ software according to the user manual. The lengths of villi (control group: 40 villi from 4 animals; MSG-induced obesity group: 40 villi from 4 animals) and thicknesses of villi (control group: 80 villi from 4 animals; MSG-induced obesity group: 80 villi from 4 animals) were measured. The volume of cytoplasm and nucleus of small intestinal epithelia from each group were also measured (control group: 80 cells from 4 animals; MSG-induced obesity group: 80 cells from 4 animals). According to the morphometric aspect of rER [22], the length of rER in cells from each group was measured (control group: 414 rER from 10 cells; MSG-induced obesity group: 415 rER from 17 cells). According to the morphometric aspect of mitochondria [22], the area of mitochondria in small intestinal epithelia from each group was measured (control group: 30 cells; MSG-induced obesity group: 30 cells). All data were expressed as mean ± standard deviation. Statistical analysis was performed using the StatView statistical software (SAS Institute Inc., Cary, NC). The differences between control mice and MSG-induced obese mice were analyzed using Student's* t*-test.* P* values < 0.05 were considered statistically significant.

## 3. Results

### 3.1. Body Weight and Macroscopic Changes in the Small Intestine

**Figure 1:**
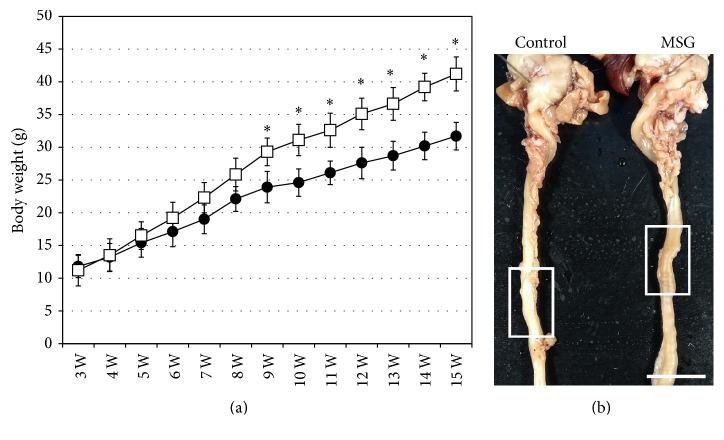
Changes in body weight and macroscopic observation of the small intestine. (a) Plot of body weight from 3 weeks to 15 weeks of age for monosodium glutamate- (MSG-) induced obese mice (white squares) and control mice (black circles). The mean ± standard deviation is shown for each group. ^*∗*^ *P* < 0.05. (b) Representative samples of the stomach and small intestine from control and MSG-induced obese mice. The white boxes indicate the area selected to investigate pathological changes. Scale bar in (b) = 1 cm.

Similar to our previous studies, obesity was induced in mice using MSG [19, 21]. In the present study, pathological changes in the small intestine of MSG-induced obese mice were examined to gain insight into changes occurring in chronic obesity in humans. MSG-induced obese mice were slightly heavier compared with control mice at 5 weeks of age, and from 9 to 15 weeks of age, the obese mice were significantly heavier (Figure 1(a)). The small intestines from the 15-week-old mice were examined macroscopically. There was no difference in length or thickness of the small intestines between the obese and control mice (Figure 1(b) and Table 1).

### 3.2. Histopathological Changes in the Small Intestine

**Figure 2:**
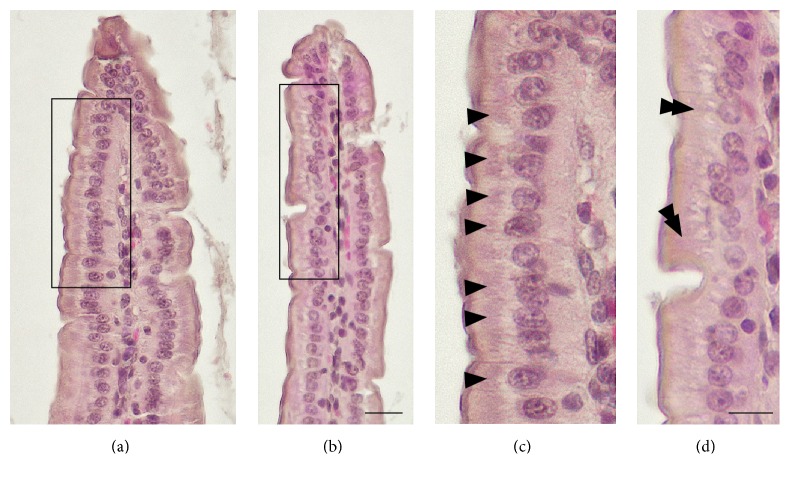
Representative light microscopy images showing histopathology of the small intestine. (a) Low magnification view of a hematoxylin-eosin- (HE-) stained section of small intestine from control mice. (b) Low magnification view of a HE-stained section of small intestine from monosodium glutamate- (MSG-) induced obese mice. Black boxes in (a) and (b) show the areas enlarged in (c) and (d), respectively. Arrowheads in (c) indicate intensely hematoxylin-stained regions in the cytoplasm. Double arrowheads in (d) indicate slight or negative hematoxylin-stained regions in the cytoplasm. Scale bars in (a) and (b) = 20 *μ*m and (c) and (d) = 10 *μ*m, respectively.

Pathological changes in the small intestinal mucosa were examined using HE-stained tissue sections (Figure 2). The tissues from control mice were healthy (Figure 2(a)). In MSG-induced obese mice, the villi were elongated compared with those from control mice (Table 2). Moreover, the villi from the obese mice were thinner compared with those from control mice (Figure 2(b) and Table 2). The single-layered epithelium in samples from MSG-induced obese mice appeared almost normal and there was no difference in the distribution and frequency of goblet cells in the epithelium between control and obese mice. In addition, there was no difference in the appearance of the central lacteals between the two groups. The mucosal epithelial cells from control mice appeared healthy, and the brush borders showed a normal structure (Figure 2(c)). There was no obvious difference between control and MSG-induced obese mice in terms of the shape and dimensions of the mucosal epithelial cells (Figure 2(d) and Table 3). However, many intensely hematoxylin-stained regions were identified in the mucosal epithelial cells from control mice (arrowheads in Figure 2(c)), but there was only slight or negative hematoxylin staining in the mucosal epithelial cells from MSG-induced obese mice (double arrowheads in Figure 2(d)). Because hematoxylin solution stains the acidic regions in cytoplasm, it was possible that the hematoxylin-stained regions were rER, which is acidic.

### 3.3. Ultrastructural Changes in the Small Intestine

**Figure 3:**
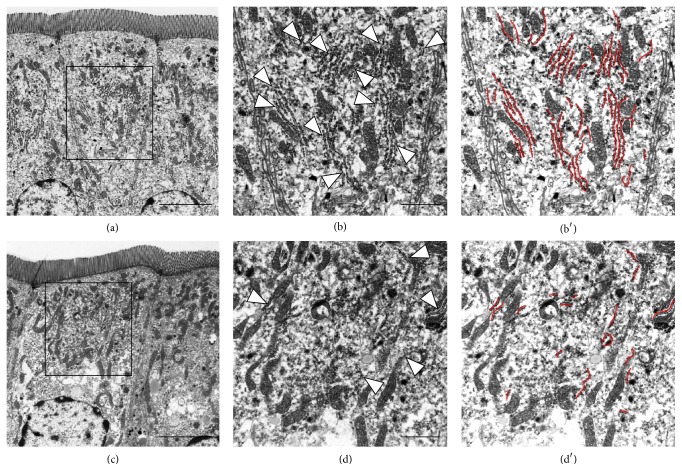
Representative electron microscopy images showing the distribution of rough endoplasmic reticulum (rER) in small intestine mucosal epithelial cells. Mucosal epithelial cells from control mice ((a) low magnification; (b) high magnification). Mucosal epithelial cells from monosodium glutamate- (MSG-) induced obese mice ((c) low magnification; (d) high magnification). Arrowheads in (b) and (d) indicate rER. Scale bars in (a) and (c) = 5 *μ*m and (b) and (d) = 2 *μ*m.

**Figure 4:**
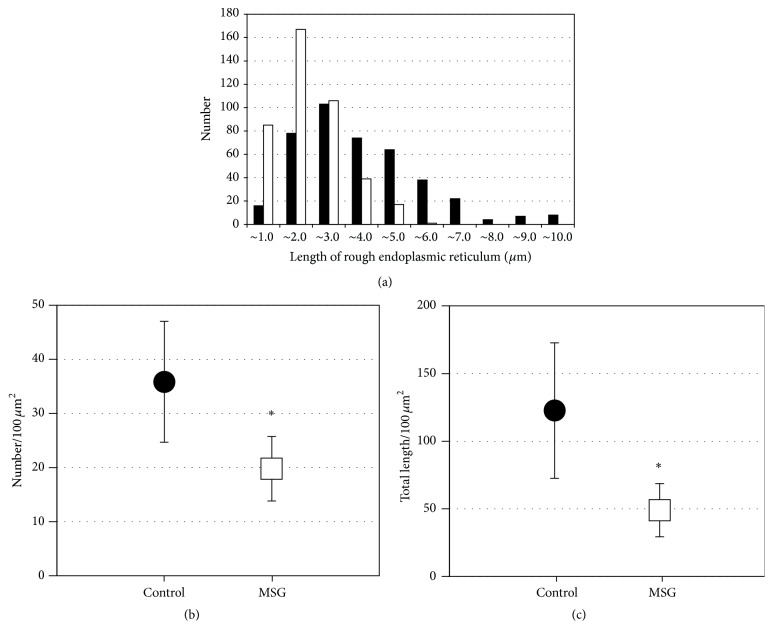
Size distribution and density of rough endoplasmic reticulum (rER) profiles measured using transmission electron microscopy. (a) Histogram of the size of individual rER profiles in small intestine mucosal epithelial cells. (b) Density of rER in small intestine mucosal epithelial cells. (c) Total length of rER in small intestine mucosal epithelial cells. The black bars and circles and the white bars and squares indicate the number of control mice and monosodium glutamate- (MSG-) induced obese mice, respectively. ^*∗*^ *P* < 0.05.

Next, pathological changes in mucosal epithelial cells were investigated using electron microscopy. On Nissl staining of sections embedded in epoxy resin, rERs in the mucosal epithelial cells of control mice were clearly detected, but few or none were detected in sections from MSG-induced obese mice (data not shown). Under electron microscopy, many rERs were detected in mucosal epithelial cells from control mice (Figure 3(a) low magnification view, Figure 3(b) high magnification view, and Figure 3(b′) rERs were highlighted by red color lines). Compared with the cells from control mice, rER was significantly decreased in cells from MSG-induced obese mice (Figure 3(c) low magnification view, Figure 3(d) high magnification view, and Figure 3(d′) rERs were highlighted by red color lines). Similar numbers of rER from both groups were measured for length using transmission electron microscopy (control 414 rER; MSG-induced obese mice 415 rER; Figure 4(a)). The length of rER in cells from control mice showed a peak at approximately 3 *μ*m. The distribution of the rER length in cells from MSG-induced obese mice peaked at a shorter length and showed a narrower distribution compared with that for control mice. Therefore, the length of rER in cells from MSG-induced obese mice was significantly shorter compared with that from control mice. The rER density in cells from MSG-induced obese mice was also significantly decreased by approximately 45% compared with that in control mice (Figure 4(b)). The average length of rER in cells from MSG-induced obese mice was significantly decreased by approximately 60% compared with that in cells from control mice (Figure 4(c)). Next we demonstrated that the volumes of mitochondria from both groups were also measured. The volumes of mitochondria from MSG-induced mice were not significantly changed compared to that of control mice (Table 4).

### 3.4. Histopathological Changes Using Histological Staining in the Small Intestine

**Figure 5:**
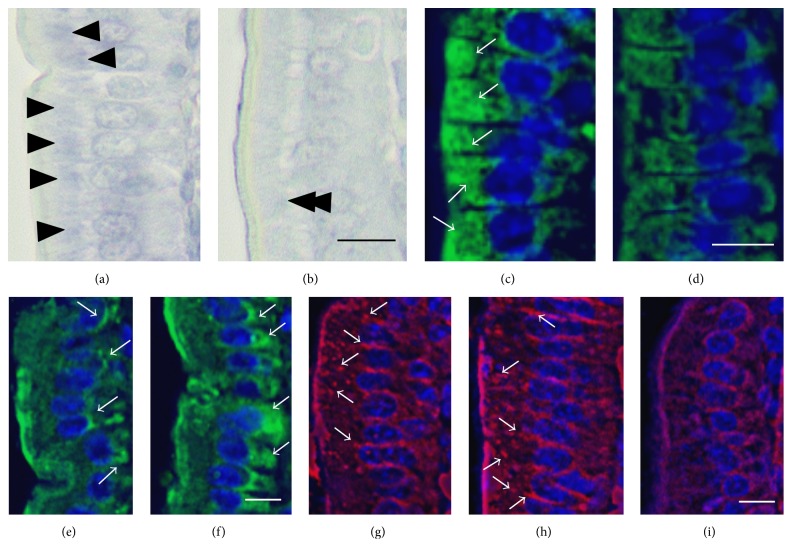
Representative light microscopy images showing the distribution of rough endoplasmic reticulum (rER) in small intestine mucosal epithelial cells. ((a) and (b)) High magnification views of Nissl-stained sections of small intestine from control mice and monosodium glutamate- (MSG-) induced obese mice, respectively. ((c) and (d)) High magnification views of rER-stained sections small intestine from control mice and MSG-induced obese mice, respectively. ((e) and (f)) High magnification views of Golgi apparatus-stained sections of small intestine from control mice and MSG-induced obese mice, respectively. (g) High magnification view of tight junction-stained sections of small intestine from control mice. ((h) and (i)) High magnification views of tight junction-stained sections of small intestine from MSG-induced obese mice. Arrowheads in (a) indicate intensely Nissl-stained regions in the cytoplasm. Double arrowheads in (b) indicate slightly or negative Nissl-stained regions in the cytoplasm. White arrows in (c) and (d) indicate rER. White arrows in (e) and (f) indicate Golgi apparatus. White arrows in (g) and (h) indicate tight junctions. All scale bars = 10 *μ*m.

To confirm whether rERs were decreased in the mucosal epithelial cells from MSG-induced obese mice, the mucosal epithelial cells embedded in paraffin were stained using Nissl solution, a basic aniline dye (Figures 5(a) and 5(b)). Nissl solution identifies rER as Nissl bodies or tigroid substance. In mucosal epithelial cells from control mice, there were many Nissl-stained regions (arrowheads in Figure 5(a)). In cells from the MSG-induced obese mice, there was only slight or negative Nissl staining in the same regions stained with hematoxylin (double arrowheads in Figure 5(b)). Moreover, the rER of mucosal epithelial cells were stained using anti-GRP78 Bip antibody (Figures 5(c) and 5(d)). In control mice, many rERs (green color) were identified (white arrows in Figure 5(c)), but there were fewer rERs in the mucosal epithelial cells of the MSG-induced obese mice compared with the controls (Figure 5(d)). And the Golgi apparatuses of mucosal epithelial cells were also stained using anti-golgin 97 antibody (Figures 5(e) and 5(f)). Many Golgi apparatuses (green color) were detected in cells from control mice (white arrows in Figure 5(e)). However, in cells from the MSG-induced obese mice, there was increased staining intensity compared with the controls (white arrows in Figure 5(f)), indicating enlargement and/or expansion of Golgi apparatus, in particular around the nucleus and basal side of the epithelial cells.

Finally, to identify the tight junction between epithelia, the mucosal epithelial cells sections were stained with anti-ZO-1 antibody (Figures 5(g)–5(i)). As previously described [23], many tight junctions were identified in between epithelia from control mice (white arrows in Figure 5(g)). In MSG-induced obesity mice, many tight junctions were also maintained in between epithelia (white arrows in Figure 5(h)); however, there were some poorly stained regions by tight junctional marker (Figure 5(i)).

## 4. Discussion

The objective of this study was to determine whether small intestinal epithelial cells show pathological changes associated with obesity. We induced obesity in mice soon after birth using MSG, as a model of obesity starting in childhood. Based on past evidence of clinical conditions in chronic obesity, we hypothesized that pathology causing functional changes in nutritional absorption and/or the integrity of the gut barrier would be observed. In human society, childhood obesity has become an increasing problem worldwide [3]. Obese children are likely to become obese adults at increased risk for many health problems [4–8]. Importantly, metabolic syndrome associated with chronic obesity is a major predictor of NAFLD, a progressive and potentially fatal disease [10, 11]. While the pathogenesis of NAFLD is partly understood, to date, little is known about structural changes in the small intestine associated with chronic obesity. Therefore, in the present study, we conducted a structural and ultrastructural investigation of the small intestinal mucosa in a mouse model of obesity immediately after birth.

While the macroscopic anatomy did not differ between control and obese mice, light microcopy identified elongated and thinned villi in the small intestine of the obese mice. It is possible that these changes could affect the function of the small intestine. In addition to the absorption of nutrients, the intestinal mucous membrane epithelium provides a defense mechanism to prevent the invasion of the pathogenic microbes such as bacteria. If this gut barrier is compromised, pathogens can pass through the intestinal mucous membrane epithelium into the blood stream. Translocation of bacteria into the portal vein is one of the causes of liver damage in NAFLD. Therefore, it is possible that functional failure of the intestinal epithelial barrier occurs in chronic obesity. Similar to the findings of the present study, the villi in diabetic model rats (streptozotocin-induced rats) and obese rats (Otsuka Long-Evans Tokushima Fatty rats) were also elongated and thin [24]. In another study using male ICR strain MSG-induced obese mice examined at 4, 8, and 12 weeks of age, the villi gradually elongated with age [25]. Therefore, elongation of the villi of the small intestine might be a change associated with obesity.

The balance between energy supply and energy consumption should be taken into consideration in the investigation of obesity. In terms of energy supply, impulsive overeating by MSG-induced obese mice has been reported in a number of studies [19, 21, 26, 27]. Body weight in these mice starts to increase compared to control mice, soon after weaning [19, 21]. It is possible that hyperplasia of the small intestine is caused by an acceleration of absorptive function. Energy consumption includes basal metabolism, thermogenesis, and physical activity. In a previous study, MSG-induced obese mice showed lower spontaneous activity compared to controls from one month of age [28]. Furthermore, growth hormone and thyroid hormone levels in MSG-induced obese mice are reduced [28–32]. The reduction of these hormones would affect energy balance by reducing basal metabolism. Therefore, in MSG-induced obese mice, obesity results from a combination of reduced energy consumption and overeating. The elongated villi in the small intestinal mucosa of obese mice observed in the present study may lead to accelerated absorption, resulting in the progression of obesity.

There was a decrease in the amount of and shortening of rER and an increase in the numbers of Golgi apparatus in the small intestinal epithelium of the MSG-induced obese mice in the present study. Moreover, many tight junctions were maintained but partly damaged in MSG-induced obesity mice. The rER has many general functions, including the folding of proteins in cisternae and the transport of synthesized proteins in vesicles to the Golgi apparatus. By limiting the passage of proinflammatory agents from the small intestine into the circulation, the barrier function of small intestine plays an important role in limiting the development of diabetes and metabolic disease. A lipoglycan-like lipopolysaccharide found on the cell surface of Gram-negative bacteria interacts with toll-like receptor 4 (TLR4) and initiates the release of proinflammatory cytokines [33, 34]. Glucagon-like peptide-1 (GLP-1) and glucagon-like peptide-2 (GLP-2) play a pivotal role in intestinal adaptation, epithelial cell proliferation, and maintenance of intestinal functions [35]. GLP-2 elicits improvements in gut permeability and endotoxemia and increases expression of zonula occludens-1 (ZO-1) and occludin [35]. ZO-1 and occludin are related to the barrier function of the intestinal mucosa [36–38]. Therefore, in addition to effects on absorptive function, a decrease in rER in the small intestinal epithelium may influence the biosynthesis of many of the proteins involved in the function of the gut barrier. Newly synthesized proteins are folded in the rER, and only properly folded proteins are transported from the rER to the Golgi apparatus. As part of the ER stress response that occurs with viral infections, folding of newly synthesized proteins is slowed leading to an increase in unfolded proteins. This ER stress is emerging as a potential cause of damage in some disorders, for example, insulin resistance in diabetes mellitus. Therefore, it is possible that a decrease in the amount of and shortening of rER in the small intestinal mucosa associated with chronic obesity could lead to dysfunction of the epithelium of the small intestine, for example, dysfunction of gut barrier by limiting the passage of proinflammatory agents, and/or the changes of nutrition absorption.

## 5. Conclusions

There was a decrease in hematoxylin-stained regions in the small intestinal epithelium of obese mice compared with controls. Furthermore, electron microscopy revealed a decrease in numbers and shortening of rER in the small intestinal epithelium of the obese mice. These results suggest that dysfunction of the small intestinal epithelium could occur in chronic obesity starting in childhood. Moreover, dysfunction of the small intestinal epithelium could affect functions such as protein synthesis and the defense mechanism that prevents the invasion of pathogenic microbes. These mechanisms could be involved in the pathogenesis of serious disorders associated with chronic obesity starting in childhood.

## Figures and Tables

**Table 1:** Macroscopic anatomical changes of small intestine at 15 weeks of age.

	Length (cm)	Weight (g)	Diameter (mm)
Control (*n* = 8)	41.7 ± 1.40	1.36 ± 0.11	2.17 ± 0.23
MSG (*n* = 8)	40.2 ± 1.24	1.39 ± 0.09	2.15 ± 0.18

The data are expressed as mean ± standard error values. MSG: monosodium glutamate.

**Table 2:** Lengths and thickness of villi in small intestine at 15 weeks of age.

	Length (*μ*m)	Thickness (*μ*m)
Control (*n* = 4)	375.70 ± 24.23	92.55 ± 8.59
MSG (*n* = 4)	394.11 ± 22.30^*∗*^	69.14 ± 9.69^*∗*^

The data are expressed as mean ± standard error values. ^*∗*^ *P* < 0.05, compared with the age-matched controls. MSG: monosodium glutamate.

**Table 3:** Volume of cytoplasm and nucleus in small intestinal epithelia at 15 weeks of age.

	Area of cytoplasm (*μ*m^2^)	Area of nucleus (*μ*m^2^)
Control (*n* = 4)	168.36 ± 39.29	21.83 ± 5.41
MSG (*n* = 4)	162.58 ± 32.25	21.66 ± 7.07

The data are expressed as mean ± standard error values. MSG: monosodium glutamate.

**Table 4:** The occupancy of mitochondria in small intestinal epithelia at 15 weeks of age.

	Occupancy of mitochondria (%)
Control (*n* = 4)	9.81 ± 0.92
MSG (*n* = 4)	10.43 ± 1.13

The data are expressed as mean ± standard error values. MSG: monosodium glutamate.
